# No evidence for binding of items to task-irrelevant backgrounds in visual working memory

**DOI:** 10.3758/s13421-017-0727-y

**Published:** 2017-06-28

**Authors:** Rob Udale, Simon Farrell, Christopher Kent

**Affiliations:** 1 School of Experimental Psychology, University of Bristol, Bristol, UK; 2 School of Psychological Science, University of Western Australia, Perth, Australia

**Keywords:** Visual working memory, Change detection, Feature binding, Location binding, Relational encoding

## Abstract

When representing visual features such as color and shape in visual working memory (VWM), participants also represent the locations of those features as a spatial configuration of the locations of those features in the display. In everyday life, we encounter objects against some background, yet it is unclear whether the configural representation in memory obligatorily constitutes the entire display, including that (often task-irrelevant) background information. In three experiments, participants completed a change detection task on color and shape; the memoranda were presented in front of uniform gray backgrounds, a textured background (Exp. 1), or a background containing location placeholders (Exps. 2 and 3). When whole-display probes were presented, changes to the objects’ locations or feature bindings impacted memory performance—implying that the spatial configuration of the probes influenced participants’ change decisions. Furthermore, when only a single item was probed, the effect of changing its location or feature bindings was either diminished or completely extinguished, implying that single probes do not necessarily elicit the entire spatial configuration. Critically, when task-irrelevant backgrounds were also presented that may have provided a spatial configuration for the single probes, the effect of location or bindings was not moderated. These findings suggest that although the spatial configuration of a display guides VWM-based recognition, this information does not necessarily always influence the decision process during change detection.

In everyday activities, items of interest will usually be encountered in the context of a background scene; for example, a keyboard and mouse are typically placed on a desk. It is not clear whether the items (keyboard and mouse) and the background (desk) are bound into a single representation, or whether the items can be represented independently of the background. Binding important items in memory to their task-irrelevant background might provide benefits to everyday visual cognition. For example, vision is suppressed while saccading between the items in a scene (Bridgeman, Hendry, & Stark, 1975). Because of this visual suppression, the visual system needs to encode the position of items to memory, in order to reestablish correspondence between the postsaccade items in the scene and the presaccade items stored in memory (Currie, McConkie, Carlson-Radvansky, & Irwin, 2000). Encoding task-irrelevant features of those items has been argued to be critical to reestablishing the correspondence of items across saccades (Hollingworth, Richard, & Luck, 2008). Additionally, information may become more or less relevant for storage over time. For example, when choosing the ripest fruit at the grocery store, one needs to search through the available options while keeping the best “candidate” in mind, to compare it to other potential candidates. However, if one is uncertain about their choice of the best candidate, it would be beneficial to keep track of previous candidates, to saccade back to them for further comparisons. As a result, an item that is irrelevant to the ongoing task at one moment may become relevant in the next, and may subsequently form part of the task-irrelevant background. This changing of relevancy over time means that items that are not currently stored in memory may need to be encoded in the near future, or their locations may need to be maintained in order to aid search for the target items.

When considering how task-relevant objects might be represented with respect to a task-irrelevant background, one relevant stream of visual working memory (VWM) research is on the encoding of relationships between items in memory and the extent to which these items are represented independently from other items in a display (Brady & Alvarez, 2011; Clevenger & Hummel, 2014; Woodman, Vogel, & Luck, 2012; Yang, Tseng, & Wu, 2015). Evidence increasingly favors a view of VWM in which individual item representations are not represented entirely independently from one another (Jiang, Olson, & Chun, 2000). According to the *relational*-*encoding hypothesis* (Yang et al., 2015), memory for individual items is encoded along with information about how they relate to other items, such as their position relative to the other items in the display (Jiang et al., 2000). The items in a display can be represented in terms of their spatial relationships to one another, which may be represented as a shape (e.g., a polygon) or as other geometric configurations. Spatial configurations have been shown to be important in more general visual cognition. For example, encoding the spatial configuration of a scene facilitates search for—and recognition of—the objects in complex scenes (Chun, 2000) and can be used to establish episodic scene memory (Hollingworth, 2007); likewise, grouping multiple moving objects into a nonrigid virtual object facilitates the tracking of those objects (Yantis, 1992).

The relational-encoding hypothesis assumes that each item in memory carries information about its relative position in this configural representation, independent of its absolute size and position (Jiang et al., 2000). Using a change detection task, Jiang et al. asked participants to study a display of colored squares. After an interval a test display was presented, and participants had to respond as to whether all the colors were changed or unchanged from the study display. Jiang et al. manipulated the locations of the squares in the test display, such that they matched the study display (in the same spatial location), were scrambled (presented in new, previously unoccupied, spatial locations), or were expanded so that the absolute positions were changed, but their relative positions to one another were the same. Jiang et al. found that scrambling the item locations between study and test reduced accuracy, relative to presenting them in their original study locations. In contrast, expanding the positions of the items in the test display, in such a way as to maintain the overall configuration, had no effect on accuracy as compared to when the locations were in the same location as in the study display. Jiang et al. proposed that change detection relies on the reinstatement of the configuration of the display but does not require memory for the absolute position of the individual items.

Some models of VWM make the additional assumption that encoding a display’s configuration is obligatory (Hayes, Nadel, & Ryan, 2007). Evidence for obligatory configuration encoding has come from change detection studies that showed an asymmetry in recognition performance between tests of spatial and visual features (Jiang et al., 2000; Campo et al., 2010). When location was task-irrelevant, changing item locations between study and test disrupted recognition based on visual features such as color and shape, and also reduced average accuracy in the task (Chen, 2009; Guérard, Morey, Lagacé, & Tremblay, 2013; Hollingworth, 2007; Hollingworth & Rasmussen, 2010; Jiang et al., 2000; Poch et al., 2010). However, when visual features were task-irrelevant, changing item features did not affect accuracy for the recognition of spatial locations (Beck, Peterson, & Vomela, 2004; Logie, 1995). This feature–location asymmetry could have occurred because remembering spatial locations might be fundamentally necessary for establishing feature binding during encoding (Logie, Brockmole, & Jaswal, 2011; Jaswal & Logie, 2011), as well as for reestablishing object correspondence during the comparison and decision stages (Flombaum & Scholl, 2006). Similar findings (Campo et al., 2010; Clark, Noudoost, & Moore, 2012; Guérard et al., 2013; Hollingworth, 2006, 2007; Hollingworth & Rasmussen, 2010; Meegan & Honsberger, 2005; Olson & Marshuetz, 2005; Treisman & Zhang, 2006; Wheeler & Treisman, 2002) have provided a wealth of evidence supporting the idea that an item’s position in the display’s configuration is obligatorily encoded in VWM and that global configuration reinstatement can be used to guide retrieval of the visual features.

There is evidence that this global configuration representation also incorporates information about the locations of task-irrelevant aspects of the display, such as the display’s background. For example, Lin and He (2012) asked participants to remember two letters that were presented at opposite ends of a task-irrelevant rectangular frame. At test, a single probe letter was presented centrally, and the rectangle was moved so that the letter appeared either in its original position, relative to the frame, or on the opposite side, relative to the frame. Recognition performance was best in the condition in which the frame was moved so that the probe appeared in its “original” relative position rather than at the opposite end of the frame, despite the probe never reappearing in its original absolute location. Furthermore, Hollingworth (2006) presented study images of computer-generated scenes containing a number of potential recognition memory targets. After a brief interval, recognition was tested for a single postcued target item. Changing the location of the target item reduced recognition performance when the original scene was presented at test (Exps. 1 and 4), but not when the target item was presented without the original scene (Exp. 4). Likewise, when the target remained in its original position, performance dropped when the nontargets changed location, relative to when the nontargets remained in their original locations (Hollingworth, 2007, Exp. 6).

One interpretation of these findings is that the participants obligatorily represented the global configuration of the entire scene, consisting of the relative locations of the target as well as the nontarget items. However, during the study display in Hollingworth’s (2006, 2007) design, the participants did not know which items they should expect to be tested on. Because participants did not know which items were later going to be probed, it would arguably have been strategically beneficial to encode all the items as if they might subsequently become targets, and in doing so encode their positions within the spatial configuration. Accordingly, this method does not tell us whether participants only encode relations between targets, or whether they preferentially—or potentially, obligatorily—encode the relations between targets and the task-irrelevant nontargets, such as the scene’s background. Furthermore, there is evidence that task-irrelevant spatial information is automatically encoded to VWM, but only when the task explicitly cues memory for location (Allen, Castellà, Ueno, Hitch, & Baddeley, 2015), suggesting that task-irrelevant spatial location is not necessarily encoded obligatorily, but might be encoded only when location is relevant to the task.

The relational-encoding hypothesis assumes that the location of each item is represented in terms of its position within the spatial configuration. However, it is still unknown whether the spatial configuration contains information only about items participants know will subsequently be probed, or whether it also includes spatial information about task-irrelevant background information. This question has implications for models of VWM, as well as models of episodic memory and scene representation, of which spatial configurations play an important role. In the experiments reported here, we examine whether people encode interitem relations between task-relevant target items and a task-irrelevant background—that is, whether the background of a scene is encoded as part of the configuration of items held in memory. If relational encoding is not strictly obligatory, and therefore background information is not utilized, changing locations, with a task-irrelevant background present, should result in memory performance similar to that when the items are presented without a background. However, if background information is encoded, changing object locations should have a detrimental effect when the objects are presented in front of a background, even if that background is minimal and task-irrelevant, relative to when the items are presented with no (or a blank) background.

To examine the binding of items to irrelevant backgrounds, we used a task based on that of Treisman and Zhang (2006, Exps. 1 and 2). In Treisman and Zhang’s study, participants completed a change detection task with three colored shapes presented in the study display. Participants were informed that the locations of the items and the bindings (the combination of a particular color with a particular shape) between color and shape were irrelevant to the task; the participants were simply to indicate whether the test display contained a new feature (color or shape). In the test display, the items could be presented at new or at their old locations, and the color–shape combinations could be maintained or switched. An example of a feature switch would be a red square and a blue circle at study becoming a red circle and a blue square at test. When whole-display probes (test displays containing all three items) were presented and the feature combinations were intact, changing locations between study and test reduced change detection accuracy, relative to keeping the locations the same. However, when the feature combinations were switched, accuracy was higher in the new-location condition than in the old-location condition. Treisman and Zhang’s interpretation of this finding was that, at least in the case of the whole-display probes, retrieving the items relied on information about the location in the test display, so that the participants did not (or were unlikely to) compare the features of an item at a particular location in the test display with the features of an item at a different location in memory. Therefore, when the test display items remained in their study locations but their features had switched, participants were more likely to decide that the features were new, despite the features originally having been presented elsewhere in the study display. This interpretation accords with relational encoding, such that when whole-display probes are presented, participants reinstate the configuration of the study display when trying to retrieve the study item features.

An important additional finding presented by Treisman and Zhang (2006) was that when single probes were presented (i.e., only one test item was displayed; Exp. 2), there was no interaction between changing locations and switching the feature combinations. Treisman and Zhang suggested that when a single item was probed, the test display did not contain the same spatial configuration (comprising the other study items) as in the study display. The interaction between location and binding, which occurs for whole-display probes but not for single probes (Treisman & Zhang, 2006), therefore provides a useful metric of the extent to which participants use the relational properties of the display in their decision process during change detection.

If participants encode the interitem relations between targets and nontarget items or backgrounds, then presenting a background would provide the opportunity to use the background as a configural cue to the location of the probed item. This would be the case even in the absence of other studied items, and therefore result in an interaction between location and binding, even for single display probes. The interaction should occur for whole-display probes irrespective of the presence of a background, because the interitem relations are always available. However, single probes are the diagnostic case, because interitem relations are not available without a background; providing a task-irrelevant background for single probes potentially enables the use of that background as a configural cue in the same way that other items do when a full display probe is presented. If participants only encode the interitem relations between items, presenting a task-irrelevant background alongside a single probe would not provide a configuration, and therefore should not produce the interaction between binding and location found by Treisman and Zhang (2006, Exp. 1) for whole-display probes.

To summarize the following experiments, in Experiment 1, we conducted a within-subjects replication of Treisman and Zhang’s (2006) experiments containing full and single probes. In addition, the stimuli were presented in either the presence or the absence of a textured background. We found an interaction between location and binding when full probes were presented, but not when single probes were displayed, irrespective of whether or not the background was available. In Experiments 2 and 3, the full-probe condition was removed, and the textured background was replaced by location markers consisting of gray squares. As in Experiment 1, Experiments 2 and 3 both revealed no interaction between the location and binding manipulations, irrespective of the presence or absence of the background markers.

## Experiment 1

Experiment 1 was an extension (within-subjects design) of Treisman and Zhang (2006) combining their whole-display probe (Exp. 1) and single-probe (Exp. 2) experiments. This allowed for direct comparison of location binding in the whole-display and single-probe contexts within the same experiment. Experiment 1 also included an additional factor: Background. The items were presented on either a neutral gray background (as in Treisman & Zhang, 2006) or on a textured background. Although the background provided some spatial context, it did not contain clearly defined items. The backgrounds were randomly generated, grayscale-textured images whose luminance values were spatially correlated following a power function (1/*f* noise) that has previously been shown to describe the statistical structure of many images in the natural environment (Field, 1987). The use of a textured background was motivated by a number of concerns. By not using a meaningful background containing everyday items, we minimized the likelihood of behavior being affected by contextual cueing effects (e.g., Chun & Jiang, 1998). In addition, in many cases in everyday life a background may be composed of a relatively homogeneous texture rather than a few clearly defined, discrete objects. For example, the texture does not contain objects with which a configuration could be created in the form of a polygon; however, it does provide a spatial pattern within which to embed the studied items. Each participant was presented the same background across all trials, and a different background was presented to each participant. The purpose of presenting the same background for all trials was to encourage binding of the items to the background. If the background was familiar and stable, we anticipated that participants would be more likely to use it as a frame of reference for binding the items to their locations.

### Method

#### Participants

Thirty-two participants (19 women, 13 men; ages 18–63 years) participated in the 2-h study for £14 (approximately $22). Because we were partly concerned with replicating the original results of Treisman and Zhang (2006), the sample size was determined using Simonsohn’s (2015) “small-telescopes” heuristic of multiplying Treisman and Zhang’s sample size by 2.5. In all three experiments, participants were recruited through online advertisements and posters in and around the University of Bristol campus, were naïve to the experimental paradigm, and reported normal or corrected-to-normal vision. Ethical approval was granted by the University of Bristol, Faculty of Science Research Ethics Committee.

#### Materials and stimuli

The stimuli were presented using MATLAB with the Psychophysics Toolbox (Brainard, 1997; Pelli, 1997), on a 17-in. TFT monitor (resolution: 1,280 × 1,024; refresh rate: 60 Hz). Participants responded via a standard USB keyboard using the “F” and “J” keys for the presence or absence of a new feature. Key–response bindings were counterbalanced across participants. Items were presented on either a uniform, medium-gray background (RGB: 128, 128, 128) or a randomly generated, grayscale-textured background whose luminance values were spatially correlated following a power function (1/*f* noise). Each participant was presented with a different texture from those given to other participants, but each participant saw the same texture throughout all trials in their instance of the experiment. The study displays consisted of conjunctions of six possible colors and shapes. The colors, which had originally been chosen for discriminability by Treisman and Zhang (2006), were red (RGB: 255, 0, 0), blue (RGB: 0, 0, 255), yellow (RGB: 255, 255, 0), green (RGB: 0, 0, 255), brown (RGB: 150, 75, 0), and violet (RGB: 238, 130, 238). The shapes were a circle, a square, an equilateral triangle, a heart, a star, and a cross. Each shape subtended approximately 60 × 60 pixels (1.89° × 1.89° visual angle, viewing distance of 50 cm). Items appeared in one of nine possible locations. The nine locations formed a 3 × 3 grid, each location consisting of 60 × 60 pixels, with 36 pixels (1.09° of visual angle) of empty space between each location. The total grid size was 252 × 252 pixels (7.62° × 7.62° visual angle). The shapes, colors, and locations were randomly chosen at the start of each trial without replacement.

#### Design and procedure

The experimental design was a fully crossed 2 (Feature: match vs. change) × 2 (Location: old vs. new) × 2 (Binding: intact vs. switched) × 2 (Probe: whole vs. single) × 2 (Background: gray vs. textured) factorial within-subjects design.

**Fig. 1:**
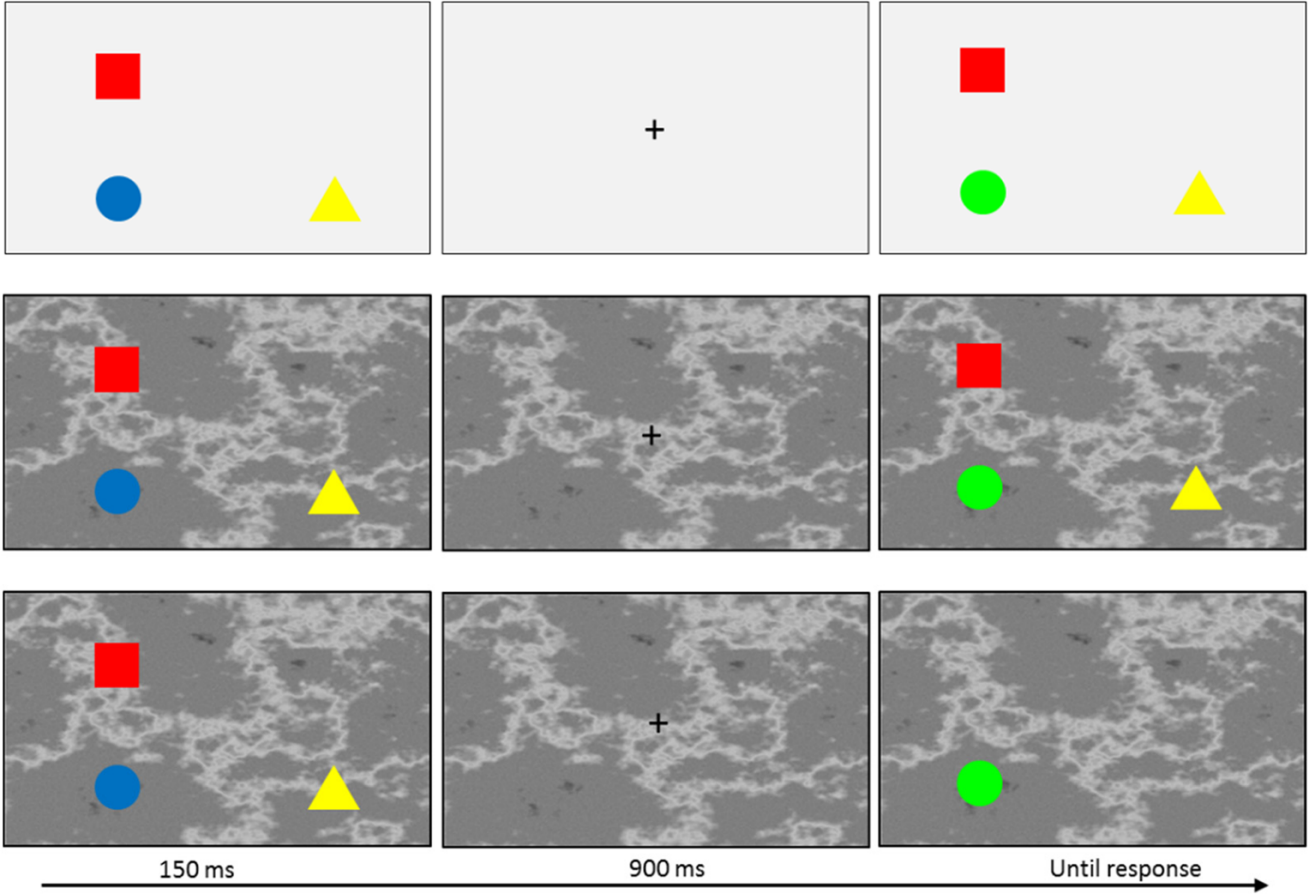
Schematic of three typical trials. These are examples of a whole-display probe with a uniform gray background (top), a whole-display probe with a textured background (middle), and a single probe with a textured background (bottom)

Participants took part in two testing sessions on different days. Each session lasted approximately 1 h. Participants took part in a practice block of 16 trials in the first session, followed by eight experimental blocks (four blocks per session). On every trial, a small white cross was presented at the center of the screen for 1,000 ms, followed by a memory display that consisted of three items shown for 150 ms. Following the study display, a blank screen with a fixation cross appeared for 900 ms. The test display was then shown and remained on the screen until a response had been made. Participants were asked to decide whether a new feature (color or shape) was present. Accuracy was emphasized over speed. Changes in location or binding were task-irrelevant, and participants were instructed to ignore them. Participants were also instructed to perform articulatory suppression by repeating “Coca-Cola” throughout each trial, to inhibit verbal strategies such as rehearsing a description of the stimuli. A schematic of typical trials from the two background conditions is shown in Fig. 1.

On half of the trials (512 out of 1,024), all of the features in the test display had been present in the study display (*match* trials), and on the other half of the trials, a new feature was present that had not been present in the study display (*change* trials). A new feature was equally often a color or a shape. On half of the trials within each block, the items in the test display kept the same color–shape binding that had been presented in the memory display (*intact* trials). On the remaining half of the trials, the binding was switched for each item in the display (*switched* trials). Color and shape switches occurred equally often. On half of the trials, the test display consisted of three items (*whole*-*probe* trials). On the other half of the trials, the test displays contained a single item (*single*-*probe* trials). On half of the trials the background was light gray throughout a block (*gray*-*background* trials), and on the remaining half of the trials, the background was textured (*textured*-*background* trials). The same background was present on screen throughout the entire testing session. The Background, Probe, and Location factors were blocked, with background being the highest level of blocking and location being the lowest level. The order in which the blocks were presented was counterbalanced across participants using a Latin-square design; the trials were blocked in this manner to remain as close as possible to the design of Treisman and Zhang (2006), which we were replicating. The Feature and Binding factors were randomized within blocks. This design, along with those of Experiments 2 and 3, is preregistered on the Open Science Framework (http://osf.io/drym6).

### Results

Although our conclusions will be primarily based on *p* values, we also analyzed the data using a Bayesian analysis of variance (ANOVA). This analysis was conducted using the anovaBF function in the BayesFactor package (Rouder, Morey, Speckman, & Province, 2012) for (R Core Team, 2015). A default Jeffreys/Zellner–Siow prior was used in all analyses, which is a Cauchy distribution with a mean of 0 and a “medium” scale of 0.5 (cf. Bayarri & García-Donato, 2007; Jeffreys, 1935, 1961; Rouder et al., 2012; Zellner & Siow, 1980). An advantage of the Bayesian approach over the frequentist approach is to assess the relative evidence favoring the null versus a specified alternative hypothesis, meaning that it is possible to measure the strength of the evidence favoring the null. The Bayes factor provides a ratio of the strengths of evidence for different models, typically an alternative model and a null model (Jeffreys, 1935, 1961). Because Bayes factors are ratios of the evidence for two models, when reported, Bayes factor indexes (either _01_ or _10_) are used to indicate which model the Bayes factor is describing. For example, a Bayes factor of 10 with the null hypothesis as the denominator (BF_10_) can also be represented as a Bayes factor of 0.1 with the alternative as the denominator (BF_01_). A Bayes factor (BF_10_) of 1 is equivocal evidence for the two models. BF_10_ corresponds to the amount of evidence in favor of the alternative over the null model. If BF_10_ is greater than 1, it is interpreted as greater evidence for the alternative, and when BF_10_ is less than 1 (i.e., BF_01_ is greater than 1), it is interpreted as evidence for the null. However, BF_10_ values between 0.33 and 3 offer only weak evidence, and BF_10_ values beyond that range can be considered as providing more substantial evidence (Jeffreys, 1935, 1961).

Before conducting data analysis, all trials with response times (RTs) below 100 ms or above 4,000 ms were removed. Less than 1% of the trials from each participant’s data (*M* = 0.68, *SD* = 0.92) were removed. Furthermore, corrected hit rates were calculated by subtracting the false alarm rate (the proportion of incorrect feature-change trials) from the hit rate (the proportion of correct feature-match trials). Participants with a mean corrected hit rate less than .1 in the baseline condition (whole-display probe, gray background, old location, and intact binding) were not included in the analysis (removing three participants); however, the qualitative pattern of results remained the same with these data included. The reason for removing participants on the basis of their performance in the baseline condition was that in some other conditions, such as the old-location, switched-binding condition, it was expected that the average performance would be near chance (see also Treisman & Zhang, 2006). Therefore, an exclusion criterion based on an average on performance in all conditions would be too strict, because it would be affected by performance in the close-to-chance conditions. Finally, on single-probe trials, it was not always possible to present the switched binding if there was also a new feature, if the switched and new features were both on the same dimension. Therefore, in the single-probe conditions, the false alarms used to calculate correct hit rates were averaged across the intact and switched bindings, similar to Treisman and Zhang’s technique. This preliminary data processing was conducted prior to analysis in all three experiments.

**Fig. 2:**
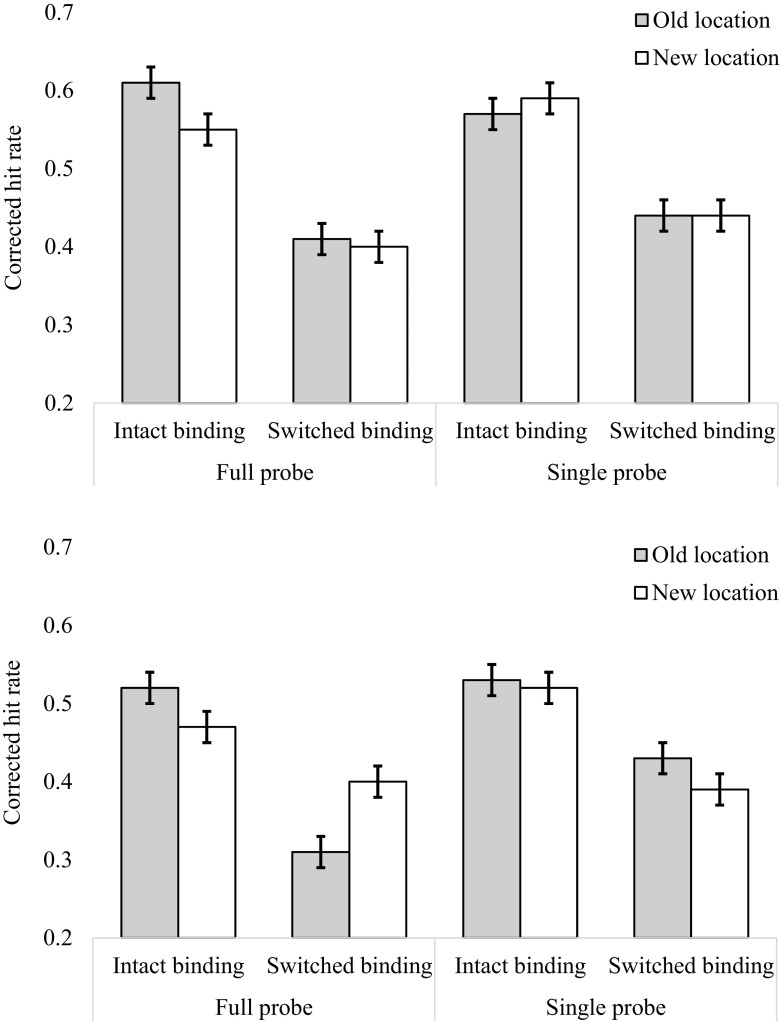
Corrected hit rates in Experiment 1 as a function of binding, location, and probe type in the gray-background condition (top) and the textured-background condition (bottom). In this and all following figures, the error bars represent within-subjects 95% confidence intervals

**Fig. 3:**
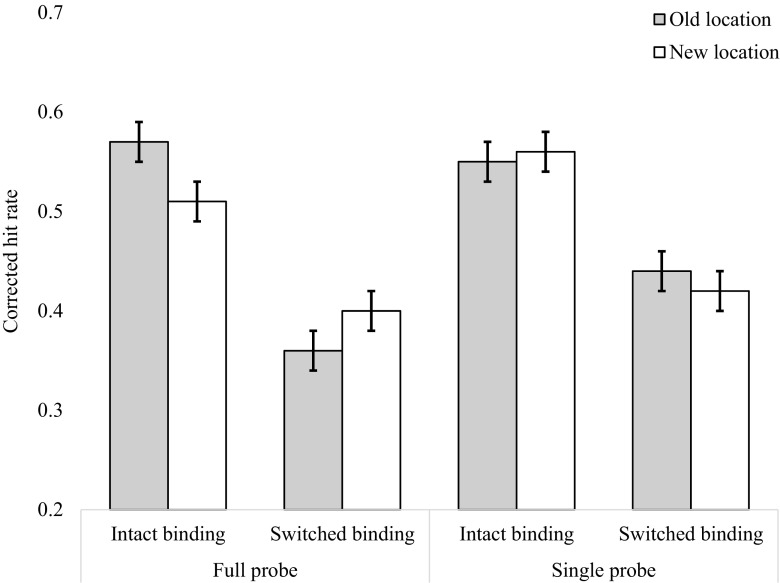
Corrected hit rates for the probe by binding by location interaction, collapsed across backgrounds

The mean corrected hit rates for each condition are shown in Fig. 2. A 2 (Background: gray vs. texture) × 2 (Probe: whole vs. single) × 2 (Location: old vs. new) × 2 (Binding: intact vs. switched) repeated measures ANOVA was conducted on the mean corrected hit rates. We found no significant main effect of probe type [*F*(1, 31) = 3.43, *p* = .075, BF_01_ = 3.7] or location [*F*(1, 31) = 0.20, *p* = .658, BF_01_ = 9.34]. The effect of background was not significant; however, the Bayes factor indicated some evidence in favor of an effect [*F*(1, 31) = 3.86, *p* = .059, BF_10_ = 3.19], with more accurate recognition with the gray background (*M* = .507, *SE* = .03) than with the textured background (*M* = .45, *SE* = .04). There was a significant main effect of binding on hit rates [*F*(1, 31) = 44.03, *p* < .001, *η*_p_^2^ = .102, BF_10_ = 1.73 × 10^9^], with participants performing more accurately when the feature bindings were intact (*M* = .55, *SE* = .04) than when the bindings were switched (*M* = .41, *SE* = .04). The three-way interaction between probe type, location, and binding was statistically significant [*F*(1, 31) = 10.14, *p* = .004, *η*_p_^2^ = .006, BF_01_ = 3.24]. All other interactions were not significant (*F*s < 3.2, BF_10_ < 3). The graph in Fig. 3 shows that when whole-display probes were presented and the bindings were intact, changing item locations reduced performance (*M* = .51, *SE* = .01) relative to keeping the locations the same (*M* = .57, *SE* = .01). In contrast, when whole-display probes were presented but the bindings were switched, changing item locations improved performance (*M* = .41, *SE* = .01), relative to keeping locations the same (*M* = .36, *SE* = .01). However, when single probes were presented, this interaction between binding and location did not occur. For single probes, the means were approximately equivalent in the old- and new-location conditions, whether the bindings were intact (old location: *M* = .55, *SE* = .03; new location: *M* = .56, *SE* = .04) or switched (old location: *M* = .44, *SE* = .03; new location: *M* = .42, *SE* = .04). The means for this three-way interaction are presented in Fig. 3. It must be noted that the *p* value suggests that there is a three-way interaction, whereas the Bayes factor indicates evidence in support of the null. This inconsistency is discussed further in the General Discussion. Finally, the four-way interaction between background, probe type, location, and binding was not statistically significant [*F*(1, 31) = 2.47, *p* = .127, BF_01_ = 9.09].

Finally, because the VWM literature has primarily focused on the assessment of change detection accuracy rather than RTs in order to estimate working memory capacity, and because our experiments are based on experiments that assessed accuracy, we were primarily interested in analyzing accuracy. However, all of the raw data, including the RT data for all experiments, are available on the Open Science Framework (http://osf.io/a93t6), as well as upon request. The RT data showed a pattern consistent with the corrected hit rate data across all three experiments. Specifically, lower accuracy was accompanied by slower RTs, and higher accuracy was accompanied by faster RTs. The Location × Binding interaction occurred for whole but not for single probes, and as with the accuracy data, this pattern was not moderated by the Background factor. From a visual assessment of the mean RT and accuracy data, no speed–accuracy trade-off appears to have occurred.

### Discussion

Experiment 1 revealed a disruptive effect of changing object locations when the bindings were intact, and a beneficial effect of changing the locations when the bindings had switched. These findings suggest that spatial information was used to guide the retrieval of the study item features when they were presented with a whole-display probe. We found that the interaction between location and binding did not occur when single probes were presented, suggesting that spatial information is not used to retrieve feature information on single-probe trials, replicating previous findings (Gilchrist & Cowan, 2014; Kondo & Saiki, 2012; Treisman & Zhang, 2006). One noteworthy finding was that we found overall equivalent performance between the two probe types. In contrast, Treisman and Zhang found higher overall performance in the single-probe relative to the whole-display probe condition. Given that this pattern was not of central interest, we do not discuss it here, but will return to it in the General Discussion.

Critically, when a textured background was presented behind the displays, the pattern of performance was effectively identical to that with a gray background. The Bayes factors provided evidence against any interactive effects involving the Background factor. Accordingly, the results provide some preliminary evidence that encoding the position of target items in relation to a task-irrelevant background is not necessarily obligatory.

One potential explanation for the lack of interactive effects of the background is that the background we used was not sufficient to allow participants to use contextual information for feature encoding and retrieval. The structure of the background was amorphous and did not contain distinct, spatially delimited areas, such as defined item placeholders. Although providing an amorphous background was intentional, it might in fact be the case that binding target items to their surrounding context must take place between specific, clearly delineated items or landmarks, which were not present in the background in Experiment 1. In Experiment 2 we investigated this further by presenting a background that comprised clearly defined locations.

## Experiment 2

In contrast to Experiment 1, for Experiment 2 we used a background composed of clearly delineated features. Previous experiments have shown that, at least in the case of whole-display probes, task-irrelevant backgrounds composed of item placeholders are sufficient to facilitate location binding (Hollingworth & Rasmussen, 2010). Hollingworth and Rasmussen showed participants four empty boxes, which were briefly filled with different colors. During the retention interval, the empty boxes either maintained their original locations or moved to new locations. At test, four colors were presented simultaneously, either in their original locations, in the locations their boxes had moved to, or in other, unrelated boxes. Accuracy was higher in the old-location and moved-location conditions, relative to the unrelated-box condition, suggesting that the participants bound the colors to their original locations as well as to the new locations the boxes had moved to. This suggests that it is possible to use external, task-irrelevant landmarks to elicit location binding, at least when whole-display probes are presented at test. Therefore, Experiment 2 replicated the single-probe conditions of Experiment 1, with the exception that the amorphous textured background of Experiment 1 was replaced with square boxes in the positions where target items could appear.

### Method

#### Participants

Thirty-two participants (21 women, 11 men; ages 18–62 years) participated for cash reimbursement (£7, approx. $11) or in exchange for course credits.

#### Stimuli

The stimuli were the same as those used in Experiment 1, except that the textured background was replaced by a medium-gray background, (RGB: 128, 128, 128) with nine dark-gray squares (RGB: 96, 96, 96) in the nine locations where stimuli could appear. The dark-gray squares were 20 pixels larger than the memoranda in both width (80 pixels) and height (80 pixels), with 16 pixels of space between the edges of adjacent squares.

#### Design and procedure

**Fig. 4:**
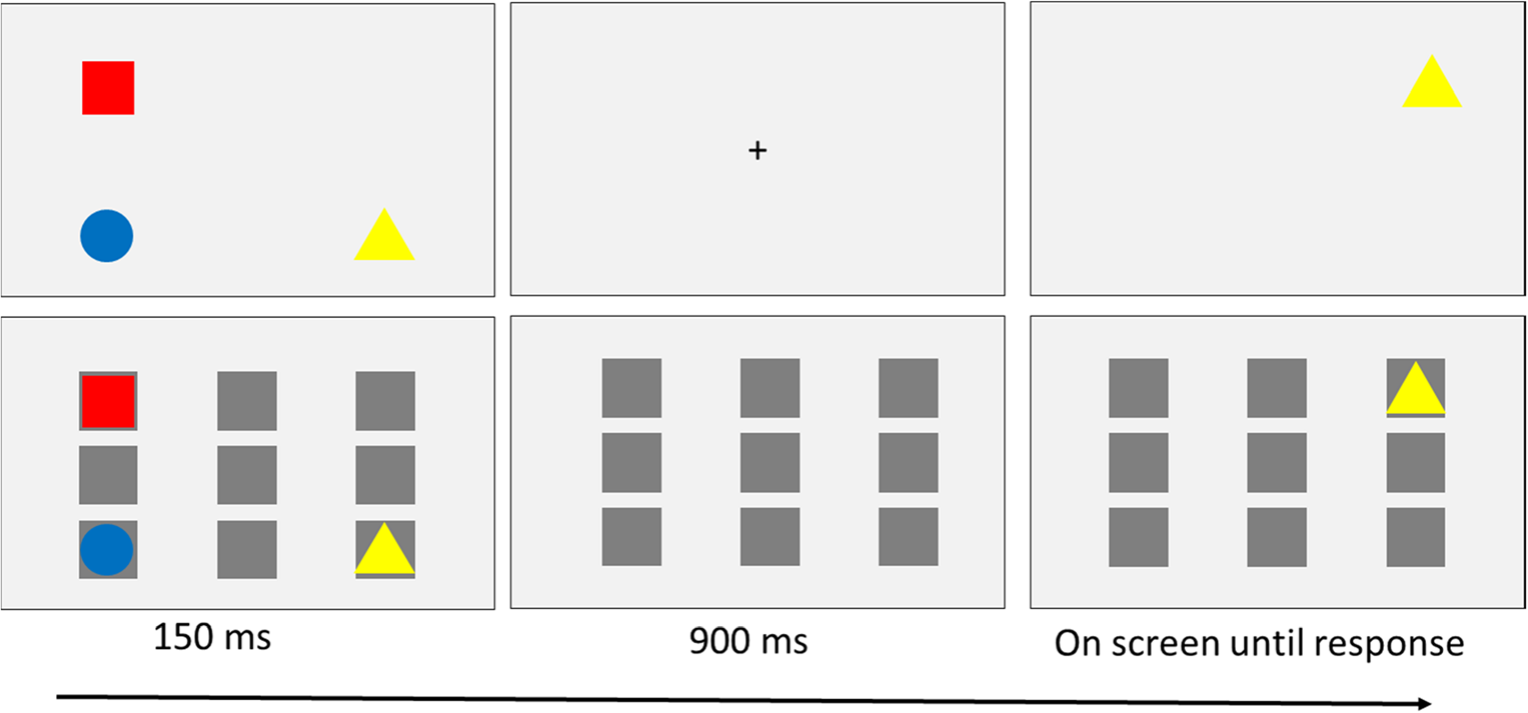
Schematic of a trial with a neutral gray background (top) and a trial with a placeholder background (bottom). Both are examples of intact-binding trials, with the test item in a new location

The design and procedure were identical to those of Experiment 1, with the exception that the whole-display probe condition was removed from the design, so that only single probes were used as the test displays. Experiment 1 had established that we could replicate the Binding × Location interaction from Treisman and Zhang (2006) in the whole-display probe condition (and not the single-probe condition). Here we were specifically interested in whether we could find evidence for a Binding × Location interaction on single-probe trials by introducing a background to encourage relational encoding and retrieval, making the whole-display probes nonessential. Thus, the experiment had a fully crossed 2 (Feature: match vs. change) × 2 (Location: old vs. new) × 2 (Binding: intact vs. switched) × 2 (Background: gray vs. squares) within-subjects design. The order of blocking in this experiment was the same as in Experiment 1, with the exception that the whole-display probe trials were removed. A schematic of typical trials from both background conditions is displayed in Fig. 4.

### Results

Prior to the analysis, the data received the same preprocessing as in Experiment 1. No participants were removed, and on average less than 1% of trials were removed per participant (*M* = 0.45, *SD* = 0.85). A 2 (Background: gray vs. squares) × 2 (Location: old vs. new) × 2 (Binding: intact vs. switch) within-subjects ANOVA was conducted on the corrected hit rates.

**Fig. 5:**
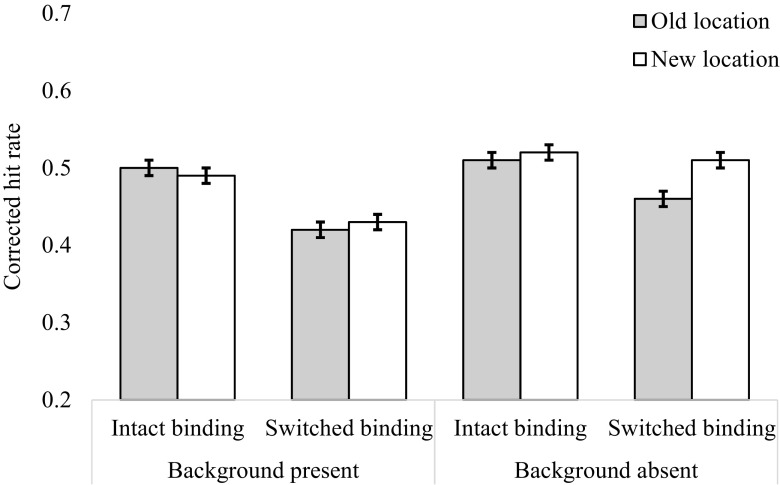
Corrected hit rates in the background, binding, and location conditions of Experiment 2

We observed a main effect of binding [*F*(1, 31) = 20.69, *p* < .001, *η*_p_^2^ = .018, BF_10_ = 1.15], in which the accuracy was higher in the intact-binding condition (*M* = .509, *SE* = .01) than in the switched-binding condition (*M* = .459, *SE* = .01). A main effect of background also emerged [*F*(1, 31) = 5.13, *p* = .031, *η*_p_^2^ = .013, BF_01_ = 1.53], in which the average accuracy was higher in the gray-background condition (*M* = .505, *SE* = .02) than in the square-background condition (*M* = .46, *SE* = .02). Note, however, that the effect sizes for both of these effects were very small, and that the BF_10_ suggested little evidence for an effect in either case. We found no main effect of location [*F*(1, 31) = 1.21, *p* = .28, BF_01_ = 5.55]. Critically, the three-way interaction between binding, location, and background also was not significant [*F*(1, 31) = 0.16, *p* = .695, BF_01_ = 7.69], nor were any of the other interactions (*F*s < 3.58, BF_10_ < 1). The mean corrected hit rates for the Background, Binding, and Location factors are presented in Fig. 5.

### Discussion

The critical difference between Experiments 1 and 2 was the type of background used. In Experiment 1, an amorphous texture was presented as the background. In Experiment 2, distinct objects (gray squares) were used as the background to mark the possible item locations. In both experiments we found no significant Location × Binding interaction. The similar results across experiments suggest that the lack of binding items to the background in Experiment 1 was not due to the amorphous nature of the background used. Thus far we have found no evidence that task-relevant items are spatially bound to their task-irrelevant background. Finally, notice that performance appeared to be lower on average in Experiment 2 than in Experiment 1. However, despite the numerical difference, we observed no evidence of a statistically reliable difference between the experiments in terms of their average corrected hit rates: Experiment 1 (*M* = .49, *SD* = .16) and Experiment 2 (*M* = .48, *SD* = .15; *t* = 0.23, *p* = .812).

## Experiment 3

One potential explanation for the lack of binding between items and locations in Experiments 1 and 2 is that perceptual grouping and segregation (Goldstein, 2009) discouraged binding of the items to their background. In Experiments 1 and 2, the backgrounds remained present throughout a trial, while the memoranda disappeared and reappeared between the study and test displays. It is possible that because the onset and offset of the backgrounds was not synchronized with the onset and offset of the study and test displays, participants were encouraged, or forced, to perceptually segment the display into a task-relevant foreground and a task-irrelevant background. A key factor in the perceptual-segmentation literature is the timing of the onsets and offsets of the background and foreground. If the foreground and background images have the same onset and offset times, they are less likely to be perceptually segregated (Kurylo, 1997). If the temporal properties of the task-relevant items match those of the background, participants may be less likely to segregate the background from those study items, and therefore be more likely to bind the two together.

Experiment 3 replicated Experiment 2, with one exception. In Experiment 2 the background squares had been present for the duration of a block. This manner of presentation may have discouraged binding of the study items and the background. In Experiment 3 the background squares were not presented during the interstimulus or intertrial intervals, meaning that their onset and offset coincided with those of the study and test display items.

### Method

#### Participants

Thirty-two naïve participants (19 women, 13 men; ages 18–26 years) took part in the study for course credits.

#### Procedure

The stimuli, design, and procedure in Experiment 3 were identical to those in Experiment 2, except that the gray squares in the background condition were only present during the study and test displays, ensuring coincidental onset and offset with the study and test items.

### Results

**Fig. 6:**
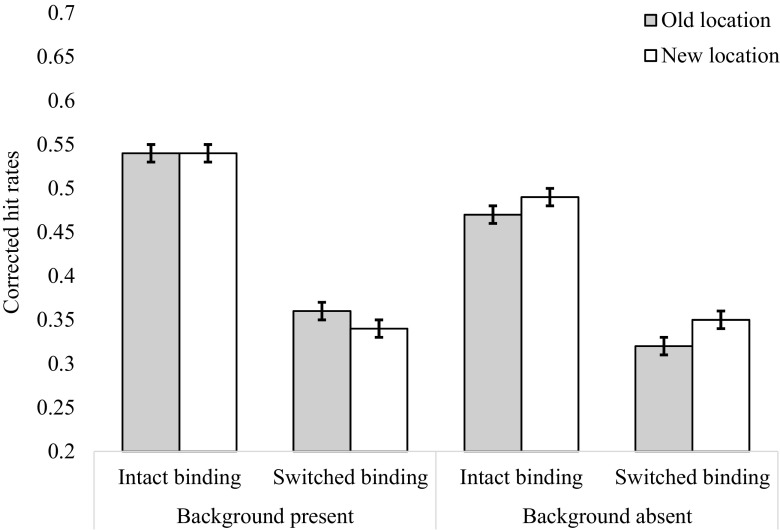
Corrected hit rates in the background, binding, and location conditions of Experiment 3

Prior to the analysis, the data received the same data processing as in Experiments 1 and 2. Three participants were removed; of the remaining 29 participants, an average of less than 1% of trials were removed (*M* = 0.56, *SD* = 0.91). The average corrected hit rates for Experiment 3 are presented in Fig. 6. A 2 (Binding: intact vs. switch) × 2 (Location: old vs. new) × 2 (Background: gray vs. squares) repeated measures ANOVA was conducted on the mean corrected hit rates. The main effect of binding was significant [*F*(1, 31) = 33.55, *p* < .001, *η*_p_^2^ = .189, BF_10_ = 1.16 × 10^9^]. On average, participants were more accurate on intact trials (*M* = .51, *SE* = .01) than on switch trials (*M* = .34, *SE* = 0.01). The main effect of background was also significant [*F*(1, 31) = 5.62, *p* = .025, *η*_p_^2^ = .012, BF_01_ = 2.43], with more accurate performance when the background was gray (*M* = .45, *SE* = .01) than when the background contained the gray placeholders (*M* = .41, *SE* = .01). The main effect of location was not significant [*F*(1, 31) = 0.04, *p* = .825, BF_01_ = 7.14].

The two-way interactions between location and binding [*F*(1, 31) = 0.08, *p* = .781, BF_01_ = 7.14] and background and location [*F*(1, 31) = 1.24, *p* = .274, BF_01_ = 5.88] were not significant. The interaction between background and binding was statistically significant [*F*(1, 31) = 4.93, *p* = .045, *η*_p_^2^ = .005, BF_01_ = 4.54]. Figure 6 shows that the effect of binding was larger when the background squares were present (intact: *M* = .54, *SE* = .01; switch: *M* = .35, *SE* = .01) than when they were absent (intact: *M* = .48, *SE* = .01, switch: *M* = .34, *SE* = .01). Critically, the three-way interaction between location, binding, and background was not significant [*F*(1, 31) = 1.14, *p* = .295, BF_01_ = 6.66].

### Discussion

The critical difference between Experiments 2 and 3 was the onset and offset of the background squares. To discourage participants from perceptually segmenting the background as being separate from the display objects, in Experiment 3 the background squares were only ever presented at the same time as the study and test displays. Despite this change in procedure, the presence of the gray squares in the background did not produce any apparent binding between items and location in reaction to a single recognition probe.

## General discussion

The primary aim of this study was to test whether change detection in VWM relies on information about the position of the target items relative to a task-irrelevant background. A number of VWM researchers have concluded that the configuration of the entire display is encoded, irrespective of whether those items are relevant to the task (e.g., Hayes et al., 2007; Hollingworth, 2007; Lin & He, 2012), or that spatial configurations play a special or fundamental role in VWM representation. For example, some have claimed that spatial configuration information may be a fundamental component of VWM (Silvis & Shapiro, 2014) and that a task-irrelevant spatial configuration is encoded effortlessly, if not automatically (Boduroglu & Shah, 2009). Vidal, Gauchou, Tallon-Baudry, and O’Regan (2005) claimed that spatial information about the whole scene is integral to representing local information and that its encoding is unrestricted by selective spatial attention. However, others have shown that visual features such as color and shape are automatically bound in VWM when they constitute intrinsic features of an object, but not when they constitute background features of a display (Ecker, Maybery, & Zimmer, 2013), and therefore the extent to which external background information is automatically encoded is a topic of ongoing debate.

**Fig. 7:**
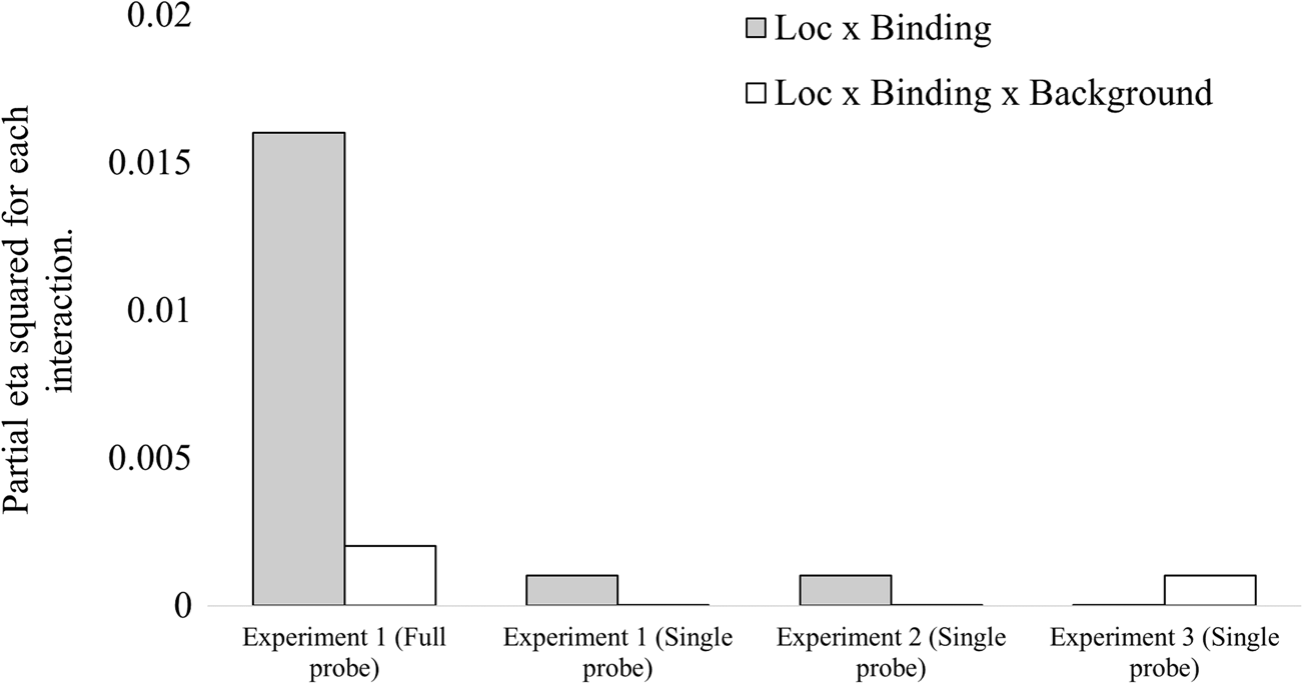
Summary of the results across all three experiments in the location, binding, and background conditions. The height of each bars represents the effect size (partial eta-squared) for each interaction

A summary of the results from the three experiments can be found in Fig. 7. Specifically, the figure shows the effect sizes (as partial eta-squared) for the Location × Binding and Location × Binding × Background interactions. As can be seen from the figure, binding items to locations occurred when full probes were presented but not when single probes were presented (as measured by the Location × Binding interaction). Additionally, this relationship was not moderated in any of the experiments by the presence or absence of a task-irrelevant background (as measured by the Location × Binding × Background interaction). In Experiment 1, when whole-display probes were presented, the participants used location to guide recognition memory, consistent with the idea that participants use the configuration of the display even when location was irrelevant to the instructed task (to detect changes in color and shape features). When single probes were presented, we found no evidence of location binding, irrespective of whether a more spatially informative background was presented (Exps. 2 and 3). If an item’s location is represented in terms of its position relative to a background, an interaction between the Location and Binding factors should have been observed for single probes, as it was for whole-display probes. However, the lack of such an effect suggests that location was not used to make recognition decisions about single probes, irrespective of the nature of the backgrounds examined here.

Previous research has shown binding between target items and task-irrelevant background items (e.g., Hollingworth, 2007; Lin & He, 2012; Sun & Gordon, 2010). For example, both Hollingworth (2007) and Sun and Gordon (2010) presented to-be-remembered scenes and cued a single target item at test, while changing the relative positions between the target and background items between study and test. In both studies, the authors found that changing the relative locations of the targets and backgrounds disrupted performance, suggesting that the target and the irrelevant background items were bound into a single representation of the display’s configuration. However, these previous findings are not necessarily in conflict with our own. During the study displays in both of Hollingworth’s studies, the participants did not know which items would subsequently become the target. Because all items could potentially become targets and because changing the relative locations of potential target items disrupts memory performance (Jiang et al., 2000; Treisman & Zhang, 2006), these items arguably were more likely to be encoded to working memory and subsequently bound to the display’s configuration. If this were the case, then it is possible that a configuration of the entire display, incorporating the task-irrelevant items, may have already been created due to uncertainty about the relevance of the task-irrelevant background items. Therefore, if the task-irrelevant items were indeed bound to the configural representation, changing their relative positions may have been more likely to subsequently disrupt memory performance. In contrast, in the design of our study, there was no uncertainty about the task relevancy of the background and foreground items—the participants knew that their memory would never be tested for information contained in the background. If item–background binding is determined by the level of uncertainty about which items will subsequently become background or foreground, participants may have been much more likely to bind the backgrounds to the spatial configuration in previous studies (Hollingworth, 2007; Sun & Gordon, 2010) than in the present study. This interpretation opens up the possibility that item–background binding may be strategic—for example, that items are bound to their background only when it is strategically beneficial to do so, such as when there is uncertainty about whether items will subsequently become foreground targets or remain “background.”

Other evidence for binding to task-irrelevant backgrounds comes from Lin and He (2012), who presented study displays of two letters inside a background rectangular frame. The test displays consisted of a single target letter, presented in the same absolute screen position across trials. However, its position relative to the frame was manipulated by moving the position of the frame around the target, so that the target appeared in either its original position (relative to the frame), or in the position of the other item (relative to the frame). Performance was better when the target was presented in its original relative position than when the target was presented in a new relative position, which is inconsistent with the findings from our experiments. This inconsistency cannot be explained by the background changing in relevancy over time, as in our experiments, the background in Lin and He’s study was irrelevant throughout the entire trial. One potential explanation for this inconsistency is that binding items to their background may involve some attentional costs, and that because the task demands in our experiment were potentially much higher than Lin and He’s, no resources were available to allow for object–background binding. For example, in our task, participants remembered three multiple-feature stimuli and were encouraged to inhibit interitem feature binding, in contrast to Lin and He’s task, which involved maintaining only two letters and for a much shorter duration. Consequently, the relatively low task demands of Lin and He’s experiment may have enabled their participants to use spare attentional capacity to bind the items to the background, whereas our participants did not have sufficient capacity or attentional resources to bind items to the background. If this is the case, then future work should test the extent to which attentional resources limit item–background binding. Specifically, item–background binding may occur only when participants have sufficient resources, such as selective spatial attention or spare working memory capacity, available to do so.

The most parsimonious conclusions from our findings are that, at least for the types of backgrounds we tested, participants do not necessarily bind target items to the task-irrelevant background, and therefore that binding items to their background is not strictly obligatory. Both our results and others’ (Blalock & Clegg, 2010; Boduroglu & Shah, 2009; Brockmole, Wang, & Irwin, 2002; Hollingworth, 2006, 2007; Jaswal & Logie, 2011; Jiang et al., 2000; Papenmeier, Huff, & Schwan, 2012; Treisman & Zhang, 2006) suggest that participants do encode the position of target items relative at least to other possible target items. However, when only one target item is present (such as in single-probe trials, in which the configuration between target items is not available), participants do not appear to rely on the position of the target item relative to the (task-irrelevant) background. This interpretation qualifies instantiations of the relational-encoding hypothesis that assume that nonspatial features are unavoidably accessed by their relative locations (e.g., Hayes et al., 2007; Hollingworth, 2007; Lin & He, 2012; Treisman & Zhang, 2006). Furthermore, it is not possible to say whether the lack of item–background binding in these experiments arose because the background was encoded, but participants chose not to utilize this information, or whether participants did not encode the background information at all. Previous studies have shown that task-irrelevant information can be encoded into VWM (Hu, Hitch, Baddeley, Zhang, & Allen, 2014), and therefore it seems possible that the backgrounds may at least have sometimes been encoded, but that this information did not enter into the decision-making process. However, using the design we have employed here, it is not possible to draw any strong conclusions about whether our conclusions relate to the encoding or retrieval of item locations.

Many models of VWM suggest that the separation of the background and target items occurs during the encoding phase. For example, the model of Hu et al. (2014) assumes that during encoding, a feature-based filter prevents the encoding of task-irrelevant features into VWM. Similarly, Jiang et al. (2000) proposed a model of the organization of VWM in which top-down and bottom-up factors, such as attention and perceptual grouping, modulate which items enter a representation of a display’s configuration. Consistent with this model, the locations of the task-irrelevant background features did not enter the configural representation, likely because the participants knew that this information was irrelevant during the study display. Answering the question of what are the minimal properties for encouraging the accessing of background–item binding will be important for further characterizing future models of relational encoding in VWM.

One finding we did not replicate was the single-probe advantage, in which accuracy is typically higher in the single-probe than in the whole-display probe condition (Treisman & Zhang, 2006; Wheeler & Treisman, 2002; Yang et al., 2015). Previously, the single-probe advantage has been detected when whole-display and single probes are presented to different participants in a between-subjects design (Treisman & Zhang, 2006; Wheeler & Treisman, 2002), but it has not arisen using a within-subjects design (Johnson, Hollingworth, & Luck, 2008). It is possible that when a within-subjects design is used and the probe types are presented in separate blocks (as in this study), participants can prepare and optimize performance for the different probe types by varying the extent to which they rely on spatial information, resulting in equivalent performance. This interpretation is supported by the finding that the single-probe advantage reappears in within-subjects designs when the two probe types are randomly intermixed across trials, making it difficult to predict the upcoming probe type and preventing participants from preparing their strategy for a particular type of probe, such as by changing the extent to which they rely on spatial information (Kondo & Saiki, 2012).

One effect observed in all three experiments was poorer performance in the background-present (vs. the background-absent) condition. In Experiment 1 the effect was not significant; however, the Bayes factor provided some marginal evidence for an effect. Additionally, Experiments 2 and 3 revealed a significant effect of background, albeit one accompanied by Bayes factors indicating ambivalence about the effect. One possible explanation for any detrimental effect of background is that the presence of the backgrounds served to disrupt configural encoding. For example, in a change detection task, Delvenne and Bruyer (2006) presented frames surrounding their stimuli and observed reduced accuracy and increased RTs. However, Delvenne and Bruyer assessed the creation of configurations between different features within the same object. Therefore, it is not clear whether this effect would also generalize to binding between discrete objects, as in our experiments. Furthermore, previous studies that have manipulated the presence or absence of background information using square placeholders have previously encouraged, rather than discouraged, configural binding (Hollingworth, 2007; Hollingworth & Rasmussen, 2010; Lin & He, 2012). An alternative explanation for the worse performance with a background may be that more perceptual clutter was on the screen when the backgrounds were present, and because the participants were not using the backgrounds, the backgrounds only served to distract participants from the task (as, e.g., concurrently presented distracting information impedes performance in tests of VWM, Allen, Baddeley, & Hitch, 2017; as well as in visual crowding, see Levi, 2008, for a review).

One caveat to our conclusions is that they are primarily drawn from analysis of the data from null-hypothesis significance testing. However, Bayesian inferential statistical approaches also provide benefits in that they can provide a measure of statistical evidence for the alternative hypothesis, as well as for the null hypothesis (e.g., Edwards, Lindman, & Savage, 1963; Rouder et al., 2012). Therefore, we supplemented classical frequentist tests with results from Bayesian ANOVA (Rouder et al., 2012). Although the two approaches pointed to the same conclusions in most cases (in particular, nonsignificant *p* values were usually accompanied by Bayes factors favoring the null hypothesis), there were several critical effects on which the two approaches provided conflicting conclusions. An interpretation based on *p* values leads to conclusions consistent with the majority of the literature on feature–location binding in VWM. That is, as well as replicating previous findings (Treisman & Zhang, 2006), we showed evidence of binding of items to locations and the role of location in maintaining features. However, an interpretation using Bayes factors suggests that, although there is strong evidence for binding between features, there is in fact evidence against binding features to locations, even in cases in which whole-display probes are presented. This is reflected in the weak evidence against the Binding × Location × Probe interaction in Experiment 1, along with strength of the evidence for a Binding × Location interaction that was at only an “anecdotal” level when whole-display probes were presented. This contradiction leaves open the possibility that location in fact does not play a substantial role in the change detection of visual features, consistent with some other previous findings (Woodman et al., 2012), and in contradiction to models of VWM that assume an important role of location in feature maintenance (e.g., Jiang et al., 2000; Olivers & Schreij, 2014; Pertzov & Husain, 2014; Rajsic & Wilson, 2014; Treisman & Zhang, 2006).

A critical factor might be expectancy about future relevance of currently irrelevant information, such that location binding might be sensitive to the statistics of the task. Expectation of task relevancy is a major determinant of which information enters VWM (Gazzaley & Nobre, 2012). For example, predictive cues that identify which aspects of a scene are task-relevant have been shown to enhance detection performance for locations, features, and objects (Chawla, Rees, & Friston, 1999; Greenberg, Esterman, Wilson, Serences, & Yantis, 2010; Kastner, Pinsk, De Weerd, Desimone, & Ungerleider, 1999; Posner, 1980). We suggest that in our experiments, the background information was not encoded to VWM because participants did not expect it to enter the decision-making stage. In cases in which the background may become relevant to decisions, it is likely to enter into the configuration guiding recognition memory (Hollingworth, 2006, 2007; Jiang et al., 2000; Kondo & Saiki, 2012).

What are the practical implications of this research? Although the implications of the research are primarily theoretical, we now have a better understanding of how foreground task-relevant information is bound to the background of the scene. In everyday life, the objects with which we work are usually presented within the context of the background of a scene. For example, icons on a computer interface are framed by the computer desktop image and the screen surround. Therefore, remembering the locations of icons in relation to the background may be beneficial. Likewise, the background may be relevant for goal-directed behavior—for instance, when we select a piece of fruit at the grocery store, the background consists of the fruit display (Vidal et al., 2005). The critical piece of practical information gained from our study is that only limited information about the background environment enters memory. Specifically, potentially relevant information appears to enter VWM, but is unlikely to be bound to the background of the scene. This has implications for critical real-world tasks such as security screening or surveillance, which involve comparing the environment (e.g., a CCTV monitor) with the contents of VWM (e.g., an image of a suspect) and filtering out task-irrelevant information. When searching for a suspect on a CCTV monitor, which may show a complex scene with many objects (e.g., people, vehicles) that change locations over time, it would be beneficial not to rely on location binding in the comparison process. Therefore, identifying contexts in which binding to the background may or may not be beneficial might be helpful in developing systems that can improve these types of tasks.

What are the implications of the results for how we conceptualize VWM? It could be argued that encoding the task-irrelevant background might be beneficial in everyday life for establishing the correspondences between items across saccades (Currie et al., 2000; Johnson et al., 2008) or directing saccades to items that are not currently relevant, but that may become relevant in the near future (Vidal et al., 2005). The results from our three experiments suggest that the position of target items is only encoded in relation to other possible target items, but not in relation to irrelevant nontarget background items. Accordingly, this view suggests that transsaccadic memory retains very little information for matching items across a saccade, and that matching is preferentially made using items that have been identified as relevant or that have otherwise been prioritized. Vidal et al. (2005) suggested that people have access to a very limited set of information in VWM, but that this information carries with it some information about the global scene. This interpretation assumes that the world acts as an external memory (O’Regan, 1992) and that the partially accessible global information could guide saccades toward information in the scene that later becomes relevant. The evidence here suggests that global scene information is not necessarily always accessible, so that searching the external memory in the world would constitute an uninformed visual search.
